# Leucine zipper-EF-hand containing transmembrane protein 1 (LETM1) forms a Ca^2+^/H^+^ antiporter

**DOI:** 10.1038/srep34174

**Published:** 2016-09-27

**Authors:** Juan Shao, Zhenglin Fu, Yanli Ji, Xiangchen Guan, Shang Guo, Zhanyu Ding, Xue Yang, Yao Cong, Yuequan Shen

**Affiliations:** 1 State Key Laboratory of Medicinal Chemical Biology, Nankai University, 94 Weijin Road, Tianjin 300071, China; 2 College of Life Sciences, Nankai University, 94 Weijin Road, Tianjin 300071, China; 3 National Center for Protein Science Shanghai, State Key Laboratory of Molecular Biology, Institute of Biochemistry and Cell Biology, Shanghai Institutes for Biological Sciences, Chinese Academy of Sciences, Shanghai 201210, China; 4 Shanghai Science Research Center, Chinese Academy of Sciences, Shanghai 201204, China; 5 Synergetic Innovation Center of Chemical Science and Engineering, 94 Weijin Road, Tianjin 300071, China

## Abstract

Leucine zipper-EF-hand-containing transmembrane protein1 (LETM1) is located in the mitochondrial inner membrane and is defective in Wolf-Hirschhorn syndrome. LETM1 contains only one transmembrane helix, but it behaves as a putative transporter. Our data shows that LETM1 knockdown or overexpression robustly increases or decreases mitochondrial Ca^2+^ level in HeLa cells, respectively. Also the residue Glu221 of mouse LETM1 is identified to be necessary for Ca^2+^ flux. The mutation of Glu221 to glutamine abolishes the Ca^2+^-transport activity of LETM1 in cells. Furthermore, the purified LETM1 exhibits Ca^2+^/H^+^ anti-transport activity, and the activity is enhanced as the proton gradient is increased. More importantly, electron microscopy studies reveal a hexameric LETM1 with a central cavity, and also, observe two different conformational states under alkaline and acidic conditions, respectively. Our results indicate that LETM1 is a Ca^2+^/H^+^ antiporter and most likely responsible for mitochondrial Ca^2+^ output.

Acting as a second messenger, calcium participates in various vital cellular processes, including muscular contraction, cell migration, fertilization and proliferation, and regulates the balance between the energy supply and demand^1^,^2^. Calcium signaling has been shown to be closely associated with the mitochondria^3^. The dysfunction of mitochondrial calcium homeostasis seriously affects physiological processes. A low concentration of Ca^2+^ in the mitochondrial matrix suppresses the activity of enzymes for ATP synthesis and the tricarboxylic acid cycle, which decreases the energy supply^4^,^5^. In contrast, sustained Ca^2+^ elevation inside the mitochondria results in an open mitochondrial permeability transition pore (mPTP) and, subsequently, cell death^6^,^7^. Therefore, the proper regulation of the Ca^2+^ concentration in the mitochondrial matrix is critical.

Mitochondrial Ca^2+^ uptake occurs primarily through the calcium uniporter complex (uniplex)^8^. The uniplex is located in the mitochondrial inner membrane and consists of five characteristic proteins that have been identified^9^, including a core channel component mitochondrial calcium uniporter (MCU)^10^,^11^, a paralog of the MCU (MCUb)^12^, two regulatory proteins of the mitochondrial calcium uptake 1/2 (MICU1/2)^13^,^14^,^15^, and the essential MCU regulator (EMRE)^16^. Recent studies added a new member into the uniplex. Mitochondrial calcium uniporter regulator 1 (MCUR1) functioned as a scaffold factor of the uniplex to mediate mitochondrial Ca^2+^ uptake^17^,^18^,^19^. In addition, there are several other possible candidates to handle mitochondrial calcium uptake^20^, such as the mitochondrial ryanodine receptor (mRyR), the rapid mode of Ca^2+^ uptake (RaM), uncoupling proteins (UCPs), the leucine zipper EF-hand containing transmembrane protein 1 (LETM1), etc.^9^.

The *LETM1* gene was first reported in 1999 and is missing in nearly all patients who suffer from Wolf-Hirschhorn Syndrome (WHS), which is a syndrome that includes severe mental and development retardation, muscle weakness and seizures^21^. LETM1 plays a critical role in maintaining the mitochondrial morphology^22^,^23^. Knocking down of LETM1 causes mitochondrial cristae to swell, whereas overexpression of LETM1 causes mitochondrial fragmenting. Additionally, LETM1 malfunction is related to cell death. It has been reported that overexpression of LETM1 in HeLa cells leads to necrotic cell death^24^. The silencing of LETM1 causes the elevation of mROS and induces autophagy^25^. LETM1 also affects cell growth and survival in *C. elegans* and in mice^26^,^27^. In *C. elegans*, the adult worms that silence LETM1 are smaller and contain fewer fertilized eggs. In the study on mice, after LETM1 depletion, their homozygous embryos died within 6.5 days, and 50% of the embryos died in heterozygosis.

The role of LETM1 in maintaining ion homeostasis has been controversial thus far. LETM1 contains an N-terminal domain (NTD) and a C-terminal domain (CTD) that are connected by one transmembrane helix. The CTD contains two EF-hand Ca^2+^ binding motifs. LETM1 was first reported as a K^+^/H^+^ exchanger^22^. However, it has been argued that LETM1 cannot act as a transporter that mediates ion flux because it only has one transmembrane helix^28^. Recently, LETM1 was proposed to possibly form a transporter via oligomerization. Moreover, as determined using a genome-wide RNA interference (RNAi) screen method in *Drosophila* S2 cells, LETM1 may function as a Ca^2+^/H^+^ antiporter^29^. LETM1 can uptake Ca^2+^ across the mitochondrial inner membrane and can extrude H^+^ simultaneously when the concentration of cytosolic Ca^2+^ is lower than 1 μM. The reconstitution of the purified protein LETM1 into the liposome showed that it can transport Ca^2+^ ions that are dependent on the H^+^ concentration^30^. However, the molecular mechanism of how LETM1 anti-transports Ca^2+^ and H^+^ still remains elusive.

In this research, for the first time, we observed that LETM1 has two conformational states and functions as a Ca^2+^/H^+^ antiporter using biochemical and cell biology methods, an *in vitro* liposome assay and negative stain electron microscopy.

## Results

### LETM1 mediates mitochondrial Ca^2+^ efflux

**Figure 1:**
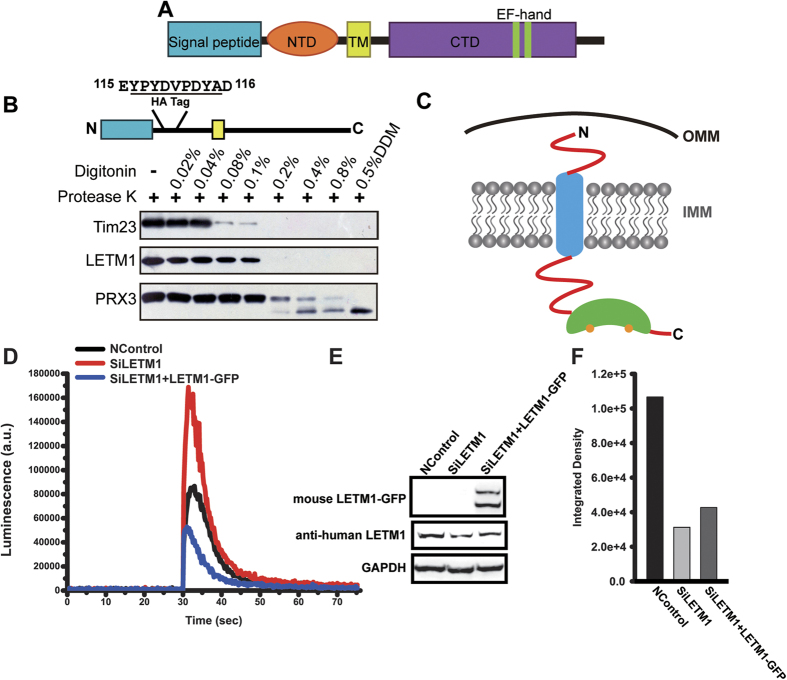
LETM1 is responsible for mitochondrial Ca^2+^ output. (**A**) Putative domain organization of LETM1. (**B**) Protease K digestion of LETM1 in mitochondria. The mitochondria were isolated from cells that overexpressed LETM1 and were incubated with increasing concentrations of digitonin in the presence of Protease K. The samples were analyzed using western blotting. An inner membrane protein TIM23, a mitochondrial matrix protein PRX3, and HA-LETM1 were tested. The HA tag position in LETM1 is indicated. (**C**) Schematic representation of the LETM1 localization on the inner mitochondrial membrane. The N-terminus of LETM1 is located on the mitochondrial intermembrane space and the C-terminus extends into the matrix. The orange sphere represents Ca^2+^. (**D**) Responses of the mitochondrial Ca^2+^ level in LETM1 knockdown HeLa cells after stimulation of 100 μM of histamine. (**E**) Western blot analysis of LETM1 protein levels in HeLa cells. Full gel and blots were shown in Figure S4. (**F**) Quantify human LETM1 protein levels only in HeLa cells by integrated density.

LETM1 has been predicted to be a single transmembrane helix membrane protein that is located in the mitochondrial inner membrane (Fig. 1A). To verify the LETM1 localization in the mitochondria, we transfected the full length mouse LETM1-GFP into HeLa cells. The result shows that mouse LETM1-GFP were co-localized with Mito Tracker Red nicely, indicating that LETM1 is localized in the mitochondria (Figure S1). To further investigate the orientation of LETM1, a protease K digestion assay was performed. HEK293T cells were transfected with full length mouse LETM1 containing a haemagglutinin (HA) tag that was inserted between Glu115 and Asp116. The mitochondria were isolated from those HEK293T cells and then incubated with different concentrations of digitonin. PRX3, a mitochondrial matrix protein and Tim23, a mitochondria inner membrane protein, were used as the controls. Our data shows that as the mitochondria inner membrane remained intact, the N-terminus of LETM1 was partially digested, indicating that the N-terminus of LETM1 was first accessed by the protease K (Fig. 1B). When PRX3 was partially degraded by the protease K, the N-terminus of HA tagged LETM1 was digested completely. Thus, it is likely that the N-terminus of LETM1 is located in the intermembrane space, and the C-terminus extends to the mitochondrial matrix (Fig. 1C). Our results are consistent with several previous reports^22^,^31^.

To further characterize the function of LETM1, we performed RNA interference experiment. HeLa cells were transfected either with small interference RNA (siRNA) which was designed to target endogenous human LETM1 to knock down (KD) its expression or with a scrambled siRNA as negative control (Ncontrol). In a comparison with Ncontrol, LETM1 KD decreased the LETM1 expression by nearly 60% at protein levels (Fig. 1E,F). Consequently, mitochondrial Ca^2+^ level was robustly elevated after stimulation with 100 μM histamine in LETM1 KD cells (Fig. 1D). While, the rescue experiment of overexpressing mouse LETM1 decreased mitochondrial Ca^2+^ level to Ncontrol levels (Figs 1D,E and S2A). Collectively, our results suggest that LETM1 is likely responsible for mitochondrial Ca^2+^ efflux.

### Glu221 plays key role in Ca^2+^ transport of LETM1

**Figure 2:**
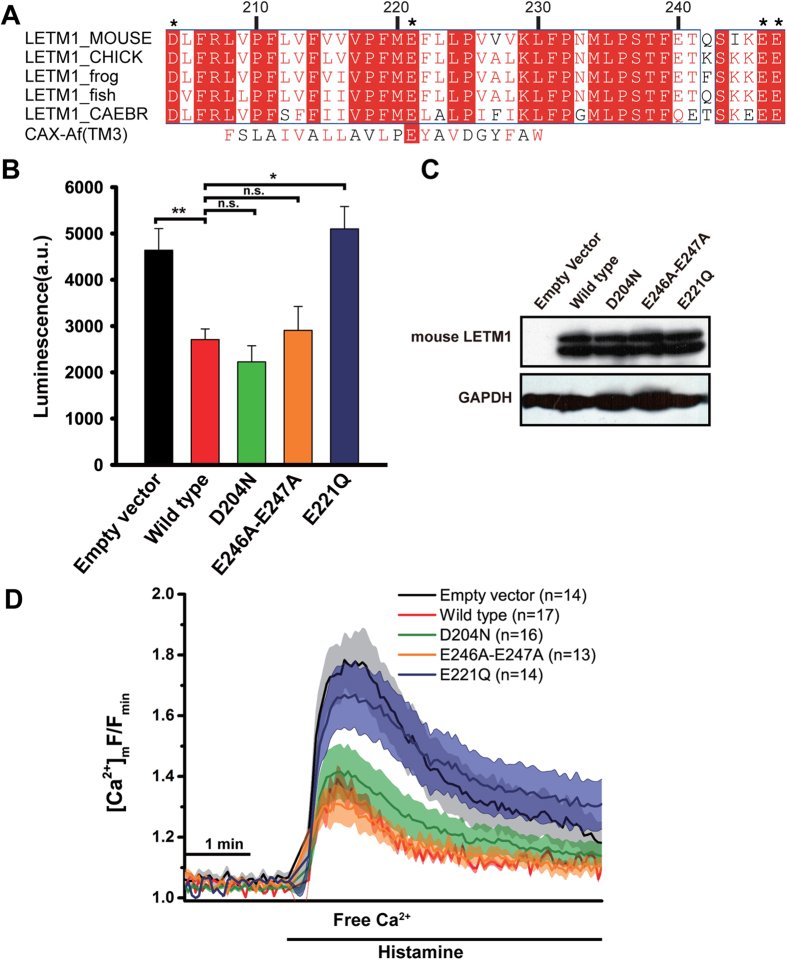
The residue E221 of LETM1 is critical for Ca^2+^ transport. (**A**) Sequence alignment of the presumed transmembrane helix. The conserved amino acids are highlighted in red. The amino acids that potentially participated in Ca^2+^ transport are designated with black stars. (**B**) Aequorin luminescence measurement. Quantification of mitochondrial Ca^2+^ peak amplitudes in empty vector, wild-type LETM1 and the mutants in HeLa cells after stimulation with 100 μM histamine. The data are expressed as the mean ± SEM, and the mean values are from 5 independent experiments. **P < 0.01, *P < 0.05, n.s, not significant. (**C**) Western-blot of the expressed LETM1 wild-type and mutants. LETM1 was immunoblotted with anti-HA antibody. Full gel and blots were shown in Figure S4. (**D**) Rhod-2 dye fluorescence measurement. Responses of the mitochondrial Ca^2+^ in the HeLa cells after stimulation of 100 μM of histamine. Solid lines represent the mean values; shaded regions represent mean ± SEM.

In general, acidic amino acids glutamates and aspartates near or in the core domain of Ca^2+^ channels or transporters may involve in mediating Ca^2+^ transport^32^,^33^,^34^. We then attempted to identify the key amino acid that is related to the Ca^2+^ transport. The LETM1 sequences from different species were aligned. Several conserved acidic amino acids (D204, E221, E246 and E247) near the predicted transmembrane helix were selected for further analysis (Fig. 2A). First, we analyzed the localization of these mutants. The LETM1 wild-type and mutant genes with a C terminal GFP tag were transfected in HeLa cells, and the results show that three mutants D204N, E246A-E247A, and E221Q were all localized in mitochondria (Figure S1). Second, we measured the mitochondrial Ca^2+^ level after overexpressing LETM1 wild type and mutants in HeLa cells with co-transfecting a mitochondria targeted aequorin mutant (mtAEQ-mut). Luminescence from the mtAEQ-mut was then counted after the histamine stimulation. Overexpression of the wild-type LETM1 significantly reduced the calcium level in the mitochondria. When compared with the wild type, overexpression of the mutants (D204N and E246A-E247A) exhibited a comparable calcium level in the mitochondria. Strikingly, overexpression of the mutant E221Q exhibited a much higher mitochondrial calcium level (Fig. 2B,C). We also verified the results using single cell-based fluorescence measurements. After transient transfection with wild-type and mutant LETM1, the HeLa cells were loaded with the mitochondrial Ca^2+^ indicator, rhod-2, and stimulated with histamine to assess the mitochondrial Ca^2+^ dynamics (Fig. 2D). As expected, the LETM1 wild type, D204N and E246A-E247A exhibited significantly lower mitochondrial Ca^2+^ levels compared with E221Q. The residue of E221, which is located in the putative transmembrane helix of LETM1, is a homologue to the residue of E78 of the transmembrane helix 3 of *Archaeoglobus fulgidus* for the H^+^/Ca^2+^ exchanger (refer to CAX_af thereafter) that is crucial for the Ca^2+^ flux^32^ (Fig. 2A). It has been noted that basal Ca^2+^ levels are similar in cells transfected by different constructs (Figure S2B,C). Therefore, LETM1 possibly behaves as a Ca^2+^ transporter that outputs Ca^2+^ from the mitochondria. Additionally, E221 may play an important role in regulating mitochondrial Ca^2+^ transport.

### LETM1 anti-transport Ca^2+^ and H^+^ *in vitro*

**Figure 3:**
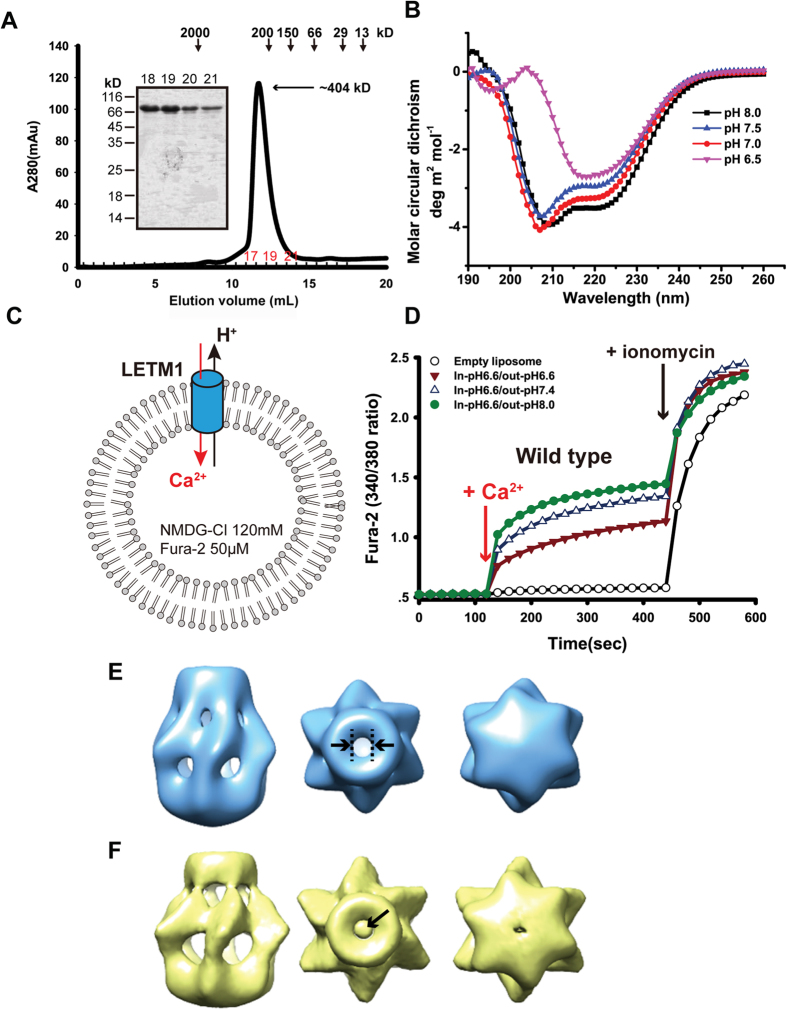
LETM1 forms a Ca^2+^/H^+^ antiporter *in vitro*. (**A**) Gel-filtration chromatography analysis of the purified LETM1. The peak fractions were analyzed via SDS-PAGE. The calculated molecular weight was approximately 404 kDa. (**B**) The pH-dependent conformational change was analyzed using circular dichroism. LETM1 exhibits an obvious conformational change when the pH shifts from alkaline to acidic. (**C**) Schematic drawing of the liposome assay system. The liposomes were prepared with 50 μM of the Ca^2+^-sensitive dye, Fura-2, inside, and the Ca^2+^ influx was initiated by adding 100 μM of CaCl_2_. (**D**) LETM1 wild-type Ca^2+^/H^+^ antiporter activities. The Ca^2+^ transport ability increased as the pH gradient increased. (**E**) Three different views of the negative staining EM density map of LETM1 at pH 8.0. The diameter of the central cavity was indicated by dotted black lines and arrows. (**F**) Views of the negative staining EM map of LETM1 at pH 6.5. The plunger was indicated by the black arrow.

To explore the possibility whether LETM1 forms a functional transporter even though it only has one transmembrane helix (Fig. 1A), we looked for additional evidence *in vitro*. We first expressed and purified the recombinant mouse LETM1 (residues 116–698) with the mitochondrial signal peptide deleted, referred as LETM1-delta hereafter. The oligomerization analysis of LETM1-delta via gel-filtration show that the purified LETM1-delta is a hexamer with a molecular weight of approximately 404 kDa (the theoretical LETM1-delta monomer is 66 kDa) (Fig. 3A). We further verified the secondary structure via circular dichroism, which surprisingly shows that LETM1 possibly exists as two conformational states under different pH conditions (Fig. 3B).

To verify the activities of recombined LETM1-delta, an *in vitro* liposome assay was conducted to measure the ion flux. The recombinant protein was incorporated into the liposome with 50 μM of Fura-2 dye. The Ca^2+^ flux was monitored by measuring the time-dependent fluorescence intensity (Fig. 3C). The recombinant LETM1-delta was found to mediate robust Ca^2+^ flux and, more importantly, the flux ability was enhanced as the pH gradient was increased (Fig. 3D). It has been noted that we fail to express the mutant E221Q as a soluble protein in *Escherichia coli* system.

To better understand the underlying mechanism of LETM1 antiport H^+^/Ca^2+^, we investigated the three-dimensional structure of recombinant LETM1-delta by negative stain electron microscopy (EM). LETM1 was purified under alkaline conditions (pH 8.0) and acidic conditions (pH 6.5), which was imaged using negative EM staining independently. The particle sizes from both the original micrograph and the reference-free 2D class averages (Figure S3) indicate that LETM1 forms a hexamer instead of existing as a monomer. Because the gel-filtration results also indicate that LETM1 may form a hexamer, we thus imposed 6-fold symmetry in the following EM data 3D reconstruction. Additionally, the structural model that was obtained at pH 8.0 shows a cavity with a diameter of 10.5 Å at the center of the hexamer (Fig. 3E, the middle panel), which is suitable for ion transportation, providing structural evidence that LETM1 can function as an ion transporter (Fig. 3E). Therefore, this conformation at pH 8.0 may represent the ion flux state. In contrast, the structure obtained at pH 6.5 shows a “plunger-like” image in which a plunger is located inside the central cavity (Fig. 3F, the middle panel), which presumably prevents ion flux. These results demonstrate that LETM1 not only forms an oligomer that acts as a transporter, but it also displays conformation transition in response to different pHs.

## Discussion

Calcium homeostasis is crucial for various cellular functions. Multiple calcium channels, transporters and receptors are involved in maintaining intracellular calcium homeostasis^2^,^9^, one of which are Ca^2+^/H^+^ antiporters. Extensive studies have confirmed that the cytoplasmic membrane of the Ca^2+^/H^+^ antiporter mediates Ca^2+^ and H^+^ exchange via an inward- to outward-facing conformational transition that is triggered by Ca^2+^ and H^+^ binding^30^,^33^. Unlike the cytoplasmic membrane of the Ca^2+^/H^+^ antiporter, the mitochondrial Ca^2+^/H^+^ antiporter candidate, LETM1, has been controversial. Our results support that LETM1 is a Ca^2+^/H^+^ antiporter.

First, the LETM1 protein without mitochondrial signal peptide was expressed and purified *in vitro*. The liposome assay indicates that the purified LETM1 is able to transport Ca^2+^, and Ca^2+^ transportation is enhanced by the proton gradient. Second, the size exclusion chromatogram shows that the purified LETM1 exists as a hexamer in solution. This is consistent with the EM studies that LETM1 hexamer images were observed under both alkaline and acidic conditions. Third, mutation of the conserved acidic residue, Glu221, significantly influence the mitochondrial Ca^2+^ level in HeLa cells, as determined via two different mitochondrial Ca^2+^ measurement methodologies. Tomohiro Nishizawa *et al.* reported that residue Glu78 is significantly involved into transporting Ca^2+^ and H^+^ in CAX_af^32^. The sequence alignment indicates that the residue, Glu221, of LETM1 may play a similar role as Glu78 of CAX_af. Therefore, our results together suggest LETM1 forms a Ca^2+^/H^+^ antiporter via oligomerization, although it only contains one transmembrane helix. It has been reported that the mechanically sensitive calcium channels, mCA1 and mCA2, also contain one transmembrane helix^35^. Oligomerization is presumably required for all these types of channels to fulfill the ion flux task.

Previous research showed that LETM1 participated in mediating mitochondria Ca^2+^ influx or efflux^25^,^29^,^30^. LETM1-mediated Ca^2+^ uptake and H^+^ extrusion occurs when the cytoplasm has a low Ca^2+^ concentration, and when the concentration of Ca^2+^ in the cytoplasm increases, the LETM1 may extrude excess Ca^2+^ to maintain the mitochondrial Ca^2+^ homeostasis^29^. The recent report showed that LETM1 may play these dual roles under different circumstances^36^. Our results showed that LETM1 knockdown by siRNA in HeLa cells results in increased mitochondrial Ca^2+^ level, while the rescue experiment by LETM1 overexpression restored the mitochondrial Ca^2+^ level to the control. Moreover, the overexpression of LETM1 wild type alone significantly decreased mitochondrial Ca^2+^ level. Also the overexpression of key residue E221Q of LETM1 showed similar mitochondrial Ca^2+^ level to the control. Taken together, our results suggest the Ca^2+^ efflux model of LETM1.

Unexpectedly, according to our biochemical and structural studies, LETM1 undergoes a conformational change under alkaline versus acidic conditions. Our EM studies show that LETM1 is oligomerized with a widely open cavity in the center at pH 8.0, which may promote Ca^2+^ transport. However, at pH 6.5, a “plunger-like” domain of LETM1 is visible in the central cavity, presumably preventing Ca^2+^ flux. The putative conformation transition regulated by the plunger-like domain seems to be similar to the working mechanism of Mg^2+^ channel MgtE^37^,^38^. Our results imply that LETM1 adopts a unique mechanism for regulating ion transportation, which presumably is different from the canonical ion transportation method of the inward- to outward-facing conformational transition^39^.

In conclusion, our findings lay the groundwork for future exploration of the LETM1 Ca^2+^/H^+^ antiporter and shed light on the fundamental aspects of mitochondrial Ca^2+^ homeostasis. Nevertheless, high resolution structures are required to elucidate the working mechanism of the LETM1 and to further address the role of LETM1 in mitochondrial Ca^2+^ homeostasis.

## Methods

### Protein expression and purification

Gene fragments that encode mouse LETM1 were amplified from the mouse cDNA library using PCR and then cloned into the pET-XMT vector, a derivative of the pET28a vector (Novagen). The recombinant protein was expressed in *E. coli*. The cells were harvested via centrifugation at 5,000 rpm for 15 min and resuspended in lysis buffer (20 mM Tris-HCl at pH 8.0, 100 mM NaCl and 1 mM phenylmethylsulfonyl fluoride). Cell debris was removed via centrifugation at 10,000 × *g* for 12 min, and the supernatant was further centrifuged to obtain the membrane at 150,000 × *g* for 90 min. The membrane was extracted for 2 hours with buffer A (20 mM Tris-HCl at pH 8.0 and 500 mM NaCl) with 1% Fos-choline 12 (Anatrace). The extract was centrifuged for 35 min at 100,000 × *g*, and then the supernatant was purified using a Ni-NTA column and a Superdex 200 size-exclusion column (GE Healthcare). The protein peak was identified using SDS-PAGE. The peak fractions were collected and used for the Ca^2+^ transport assay.

### Amphipol exchange and detergent removal

The LETM1 protein was mixed with Amphipol (Anatrace) in a 1:3 (*w/w*) ratio, and the mixture was rotated gently using a vertical mixer for 4 hours at 4 °C. The detergent was removed using three batches of Bio-Beads SM-2 (Bio-Rad). Typically, 200 mg of Bio-Beads per 1 mL of a protein/detergent/amphipol mixture was used. The first two batches were rotated for 1 hour, and the third batch was rotated overnight at 4 °C^40^. The beads were removed via centrifugation at 3,000 rpm for 5 min before further separation on a Superdex-200 equilibrated with a buffer that consisted of 20 mM Tris at pH 8.0, 500 mM NaCl, and 2 mM DTT. The peak fraction was collected for analysis with negative staining electron microscopy.

### Cell culture and transfection

The HEK293T and HeLa cells were cultured in Dulbecco’s modified Eagle’s medium (DMEM, Sigma-Aldrich, Co.) that was supplemented with 10% fetal bovine serum (FBS, HyClone Thermo Fisher Scientific, Inc.). The cells were cultured in a 95% air and 5% CO_2_ environment at 37 °C.

Gene fragments that encoded the full length of the mouse LETM1, E221Q, E246A-E247A, and D204N were cloned into vector pcDNA4/Myc-His (Invitrogen). A haemagglutinin (HA) tag was inserted between the Glu115 and Asp116 residues of the mouse LETM1 using a standard PCR-basal mutagenesis method, and the accuracy of the plasmid was confirmed by DNA sequencing.

The cells were transfected with polyethylenimine (PEI) according to the manufacturer’s instructions.

### Mitochondria isolation

After transfection for 24 hours, the HEK293T cells were collected in PBS buffer that contained 136 mM NaCl, 2.7 mM KCl, 10 mM Na_2_HPO_4_ and 1.5 mM KH_2_PO_4_ at pH 7.4. Then, the cells were harvested via centrifugation at 600 × *g* for 10 min and resuspended with a mito-buffer containing 20 mM HEPES at pH 7.5, 200 mM sucrose, 1 mM EDTA, 1 mM EGTA and 10 mM KCl. After gentle homogenization with a Dounce homogenizer, the cell lysates were removed via centrifugation at 600 × *g* for 10 min. The supernatant was then centrifuged for 15 min at 7,000 × *g* to obtain the mitochondria.

### Protease K digestion and western blot analysis

The isolated mitochondria were suspended in mito-buffer and treated with digitonin at different concentrations or with 0.5% n-dodecyl-β-maltoside (DDM) in addition to 100 μg/ml of Protease K on ice for 30 min. The digestion was terminated by adding 5 mM of phenylmethylsulphonyl fluoride (PMSF, Sigma-Aldrich). The mitochondrial proteins were separated using SDS-PAGE and transferred onto a PVFD membrane (Millipore) for western blot analysis.

### RNA interference and cDNA rescue experiments

HeLa cells were cultured in DMEM with 10% FBS and were plated onto a 24-well plate (Costar). For RNA interference, 20 nM siRNA targeted to human LETM1 was transiently transfected into the HeLa cells with Lipofectamine RNAiMax (Invitrogen) according to the manufacturer’s instructions. Specific siRNA oligonucleotides (Ribobio) for human LETM1 were targeted against the following sequences: 5′-AAUACGUGGAAGAAUCUAA-3′, which is used in previous reports^25^. The control siRNA oligonucleotide was obtained from Ribobio.

For LETM1 rescue test, the LETM1 knockdown HeLa cells were co-transfected with mouse LETM1 which is resistant to human LETM1 siRNA and a mitochondria targeted mutated aequorin (mt-AEQ_mut_) using Polyethylenimine (PEI) after 24 h of siRNA transfection. Knockdown and rescue efficiency was tested by Western-blot. The antibody to detect human LETM1 was purchased in Abcam with catalog number ab173109.

### Aequorin luminescence measurement

The transfected cells were loaded with 2 μM coelenterazine-hcp in Ringer’s buffer, which contained 135 mM NaCl, 5 mM KCl, 0.4 mM KH_2_PO_4_, 20 mM HEPES and 55 mM glucose at pH 7.4, for 2 hours at room temperature, and then they were washed twice with Ringer’s buffer. After 30 seconds of baseline recording, 100 μM of histamine were challenged. The luminescence was measured using a SpectraMax i3x (Molecular Devices) microplate reader at 460 nm every 0.2 s.

### Rhod-2 fluorescence measurement

Cells grown on a 20-mm glass-bottom cell culture dish were incubated for 50 min at room temperature in Ringer’s buffer that was supplemented with 2 μM Rhod-2/AM, and then they were reloaded with dye-free Ringer’s buffer for another 30 min. The cells were stimulated using 100 μM of histamine to mobilize the Ca^2+^ at the 30th image of recording. Images were recorded at 560 nm excitation every 3 s using a DMI-6000D (Leica) microscopy.

### Liposome reconstitution and Ca^2+^ transport assay

To test Ca^2+^ transport ability of the LETM1 protein, the purified LETM1 was incorporated into a liposome via a method previously described with a few modifications^41^,^42^. Briefly, a lipid mixture of POPE and POPG (1:3) was dissolved with chloroform and dried under an argon stream for 30 min at room temperature. The dried lipid was suspended in reconstituted buffer that contained 50 mM HEPES at pH 6.6, 120 mM NMDG-Cl and 50 μM Fura-2. The suspension was sonicated to transparency, followed by five cycles of freeze-and-thaw. After extrusion 11 times through a 400 nm polycarbonate filter (Avanti), the liposomes were destabilized with 1.0% n-octyl-α-D-glucoside (OG) (Anatrace) for 15 min at 4 °C. Then, the purified LETM1 protein was added to the liposome at a protein-to-lipid ratio of 1:100 (*w/w*). The detergent was removed using Bio-Beads SM-2 as described above.

The proteoliposomes were harvested via centrifugation at 200,000 × *g* for 40 min and suspended in a flux assay buffer that contained 50 mM HEPES at pH 6.6, pH 7.4 or pH 8.0 and 120 mM of NMDG-Cl. The Ca^2+^ flux was initiated by adding 100 μM of CaCl_2_. The fluorescence intensity was monitored using a Synergy4 spectrophotometer (BioTek) every 20 s at an emission wavelength of 510 nm and at an examination wavelength of 340 nm or 380 nm. To analyze the result, the fluorescence intensity was normalized to a ratio of 340/380 nm.

### Electron microscopy data collection

The LETM1 protein at pH 6.5 or pH 8.0 was analyzed via negative staining electron microscopy in the same manner. The sample was prepared by adding 5 μl of an amphipol-exchanged protein sample that was diluted to approximately 10 μg/mL to a glow-discharged 400-mesh continuous carbon grid (Beijing Zhongjingkeyi Technology). Then, the sample was stained with 0.75% (w/v) uranyl formate and air dried. Negatively stained EM grids were imaged on a Tecnai G2 F20 TWIN transmission electron microscope (FEI Company, USA) that is equipped with a field-emission gun, which operates at 200 kV. Images were recorded at a nominal magnification of 80,000× with a 4 k × 4 k Eagle CCD camera, which correspond to a pixel size of 1.15 Å/pixel on the specimen with a defocus ranging from −0.6 to −1 μm. Tilt pair images for the random conical tilt (RCT) 3D reconstruction were manually recorded at 45° and 0°, respectively.

### Electron microscopy image processing and three-dimensional reconstruction

For the 3D reconstruction, 52,903 particles and 28,567 particles were boxed out for the LETM1 at pH 6.5 and pH 8.0, respectively, utilizing the e2boxer.py program in EMAN2.1^43^. The reference-free two-dimensional analysis was conducted using the EMAN 1.9 program, refine2d.py^44^,^45^. A RCT 3D reconstruction was conducted for the initial model generation. For this process, e2RCTboxer.py was used to pick particles from the tilt pair images and to determine the tilt axis and angles. The 3D reconstruction was performed using the EMAN1.9 program, which was refined with the imposed 6-fold symmetry. The resolution was estimated to be 15 Å and 17 Å for pH 6.5 and pH 8.0 state, respectively, using the 0.5 FSC criteria and the *eotest* program in EMAN1.9. We used UCSF Chimera (http://www.cgl.ucsf.edu/chimera/) to render the electron microscopy density map.

## Additional Information

**How to cite this article**: Shao, J. *et al.* Leucine zipper-EF-hand containing transmembrane protein 1 (LETM1) forms a Ca^2+^/H^+^ antiporter. *Sci. Rep.* **6**, 34174; doi: 10.1038/srep34174 (2016).

## Supplementary Material

Supplementary Information
