# Supplementary material for: Leucine zipper-EF-hand containing transmembrane protein 1 (LETM1) forms a Ca2+/H+ antiporter
Source: Sci Rep. 2016 Sep 27;6:34174. doi: 10.1038/srep34174 (PMC5037442; doi:10.1038/srep34174)

Supplementary Information

Leucine zipper-EF-hand containing transmembrane protein 1 (LETM1) forms a Ca^{2+}/H^{+} antiporter

Juan Shao[1,2,6], Zhenglin Fu[3,4,6], Yanli Ji[1,2], Xiangchen Guan[1], Shang Guo[1], Zhanyu Ding[3,4], Xue Yang[1,7], Yao Cong[3,4,6], Yuequan Shen[1,2,5,7]

[1]State Key Laboratory of Medicinal Chemical Biology, Nankai University, 94 Weijin Road, Tianjin 300071, China; [2]College of Life Sciences, Nankai University, 94 Weijin Road, Tianjin 300071, China; [3]National Center for Protein Science Shanghai, State Key Laboratory of Molecular Biology, Institute of Biochemistry and Cell Biology, Shanghai Institutes for Biological Sciences, Chinese Academy of Sciences, Shanghai 201210, China; [4]Shanghai Science Research Center, Chinese Academy of Sciences, Shanghai 201204, China; [5]Synergetic Innovation Center of Chemical Science and Engineering, 94 Weijin Road, Tianjin 300071, China

[6]Contribute equally

[7]To whom correspondence may be addressed.

Xue Yang, E-mail: yangxue@nankai.edu.cn; Tel: +86-22-23504757

Yao Cong, E-mail: cong@sibcb.ac.cn; Tel: + 86-21-2077-8199

Yuequan Shen, E-mail: yuequan74@yahoo.com; yshen@nankai.edu.cn; Tel: +86-22-23506462

Supplemental Figure 1 Localization of wild-type and mutant LETM1 in HeLa cells.

Supplemental Figure 2 Mitochondrial Ca^{2+} level measurement.

Supplemental Figure 3 Negative staining EM map of LETM1.

Supplemental Figure 4 Full length gel and blots.

Supplementary Figure 1

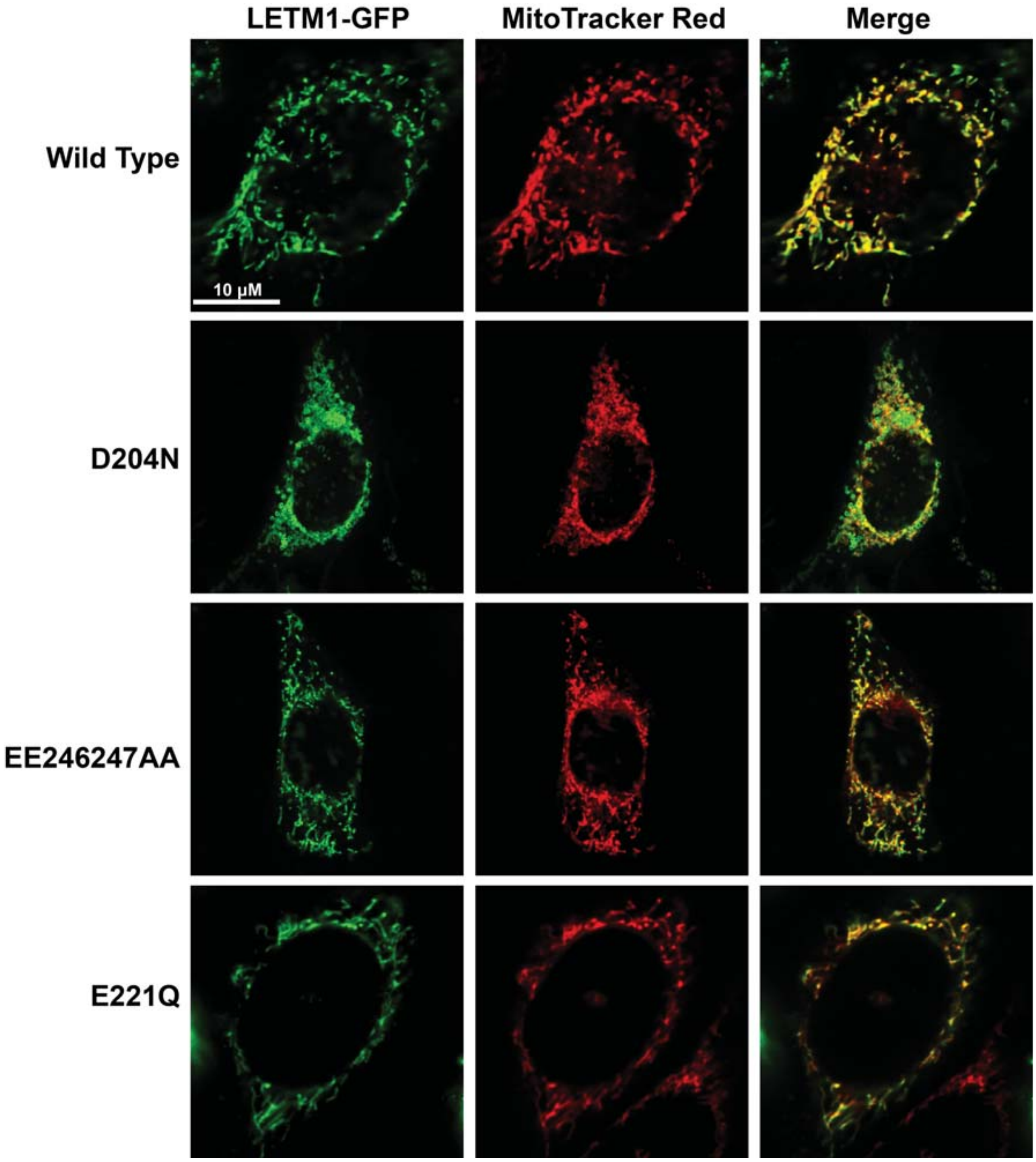

Localization of wild-type and mutant LETM1 were transfected into HeLa cell. All LETM1 constructs were C-terminally tagged with EGFP (first image in each row). Transfected cells were then treated with MitoTracker Red (second image in each row).
Scale bars are 10 μm.

Supplementary Figure 2

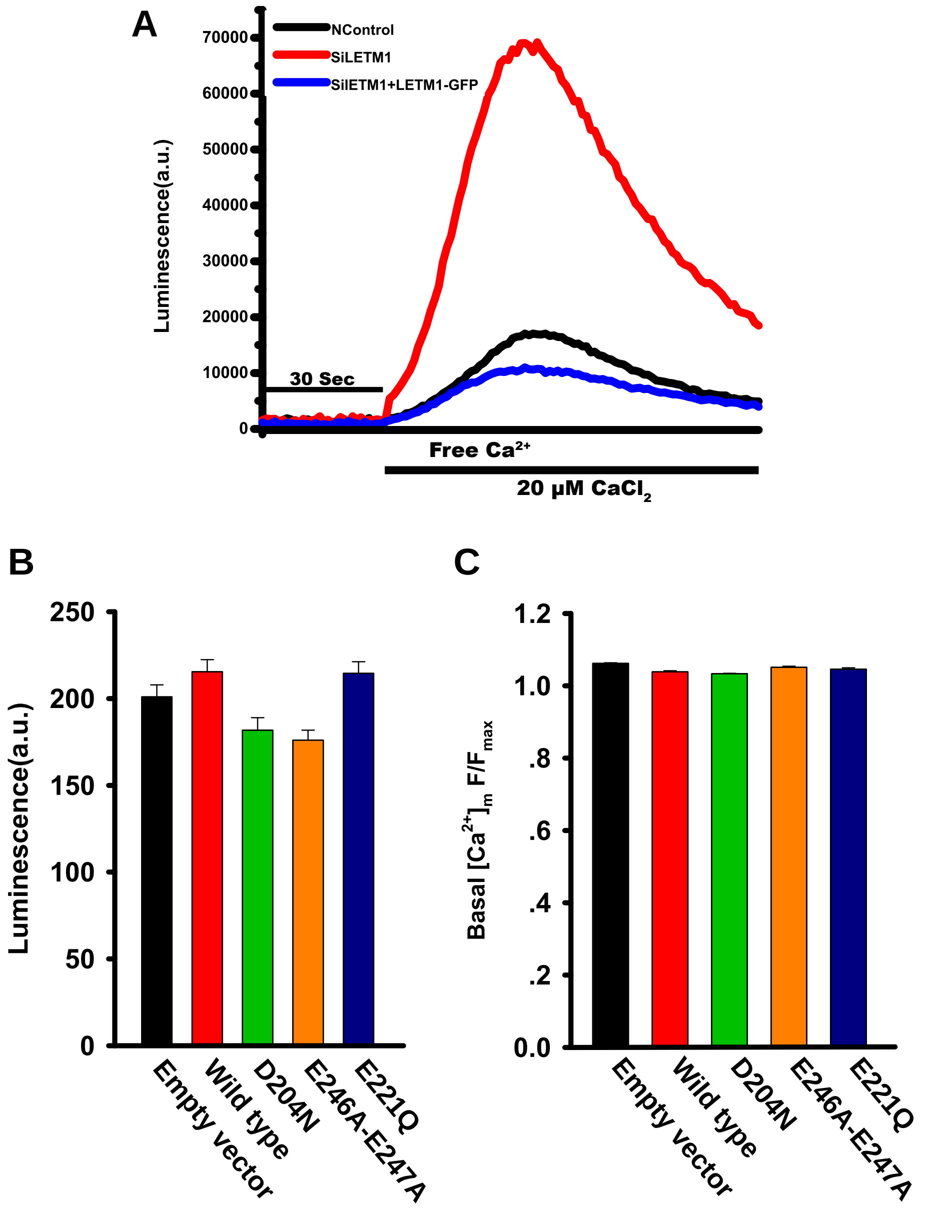

Mitochondrial calcium measurement. (**A**) Average traces of mitochondrial Ca^{2+} uptake profile in Negative siRNA (Ncontrol), LETM1 knockdown (SiLETM1) and mouse LETM1 rescue (SiLETM 1+LETM1-GFP) transfected permeabilized HeLa cells after challenging with 20 μM Ca^{2+} pulses. Briefly, The transfected cells were loaded with 2 μM coelenterazine hcp for 2 h in Ringer's buffer at room temperature. The cells were then bathed in intracellular-like buffer (ICB, 120 mM KCl, 1 mM KH2PO4 , 5 mM succinate, 50 mM HEPES, pH 7.4, 1 mM EGTA) after permeabilizing by ICB supplemented with 20 μg/mL digitonin. After 30 seconds of baseline recording, ICB was exchanged for ICB contained 20 μM free Ca^{2+}, the luminescence intensity was measured by Synergy4 spectrophotometer (BioTek) at 460 nm every 1 s. Basal calcium level measurement by Aequorin luminescence (**B**) and Rhod-2 dye fluorescence (**C**).

Supplementary Figure 3

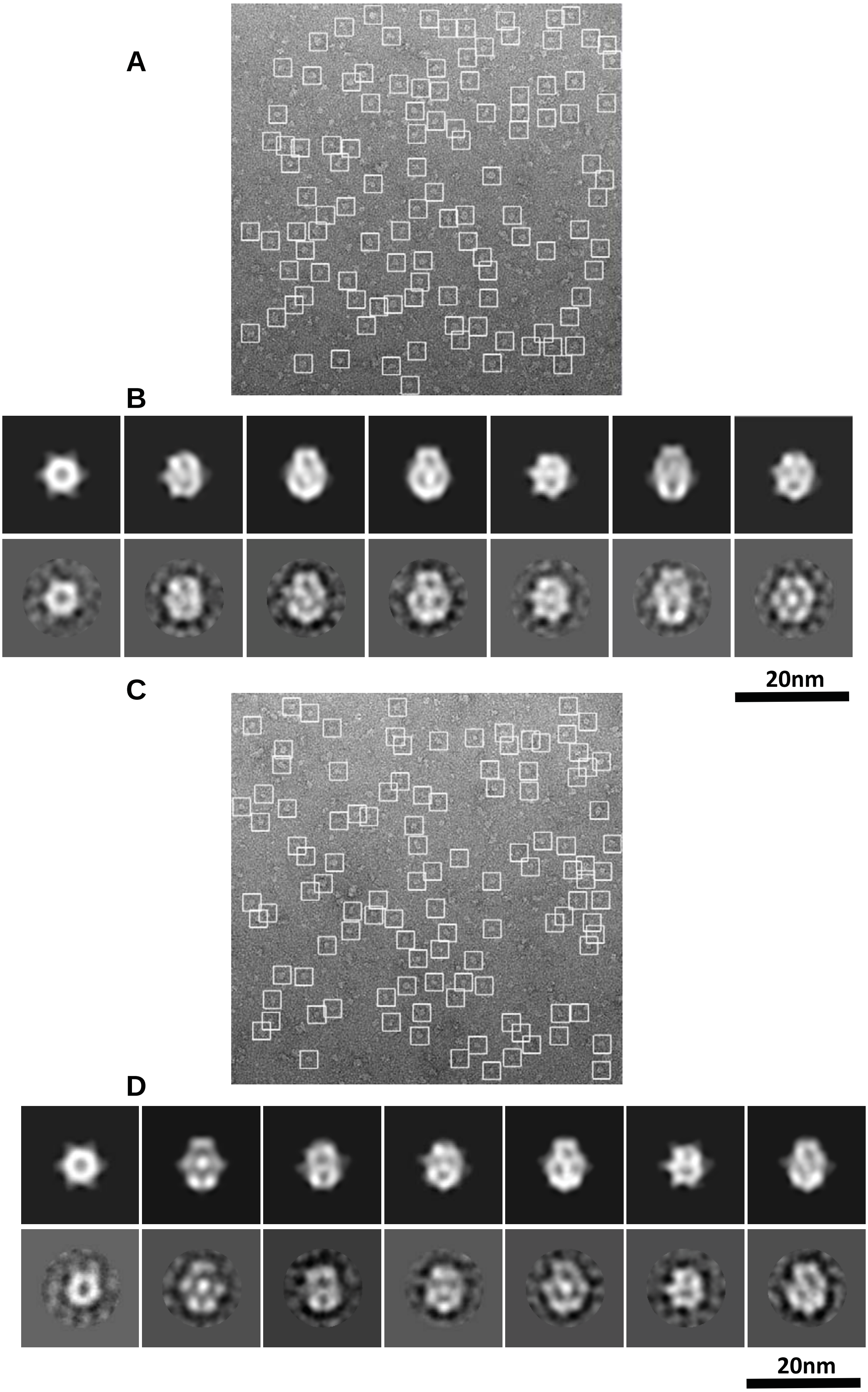

Negative staining EM map of LETM1. Typical negative stain CCD images of LETM1 in pH 8.0 (A) and pH 6.5 (C). Representative particles are highlighted by white boxes. The comparison of the 2D projections (top row) from the negative staining EM density map of LETM1 at pH 8.0 (B) and pH 6.5 (D), with the corresponding reference-free 2D class averages (bottom row) reveals similar structural features.

Supplemental Figure 4

Full-length Gel and Blots

Figure 1B

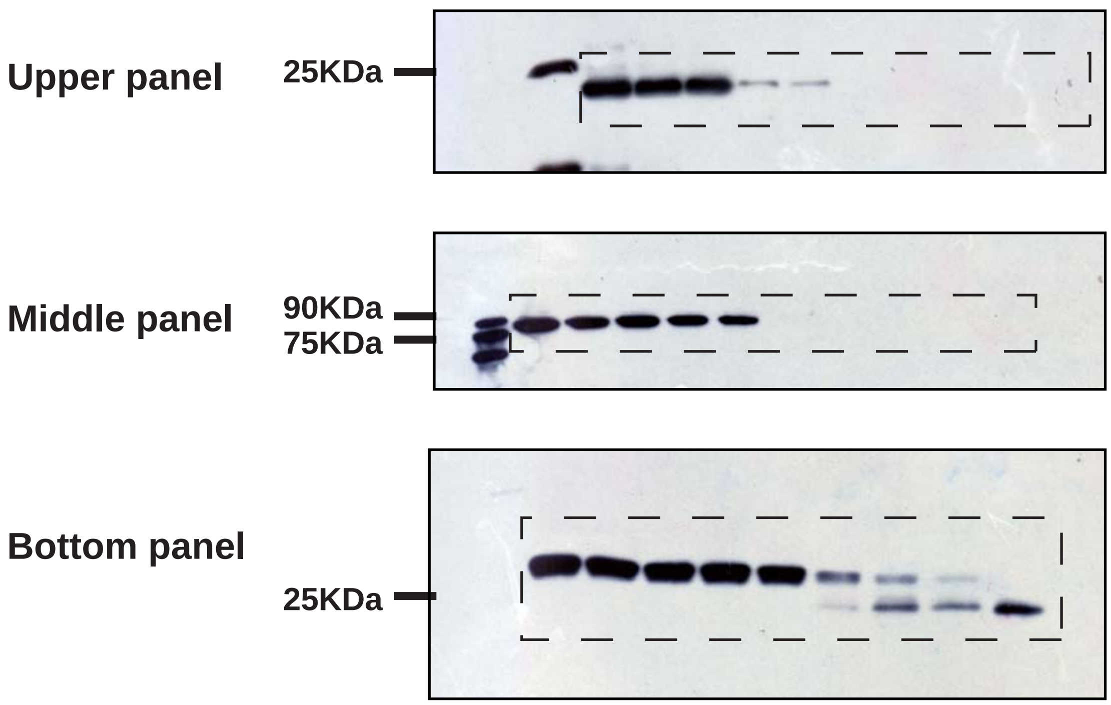

Figure 1E

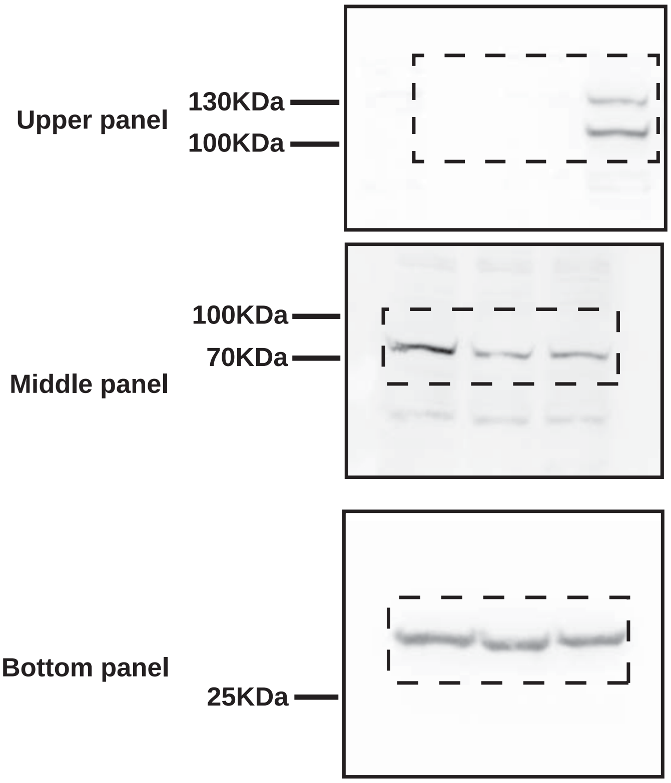

Figure 2C

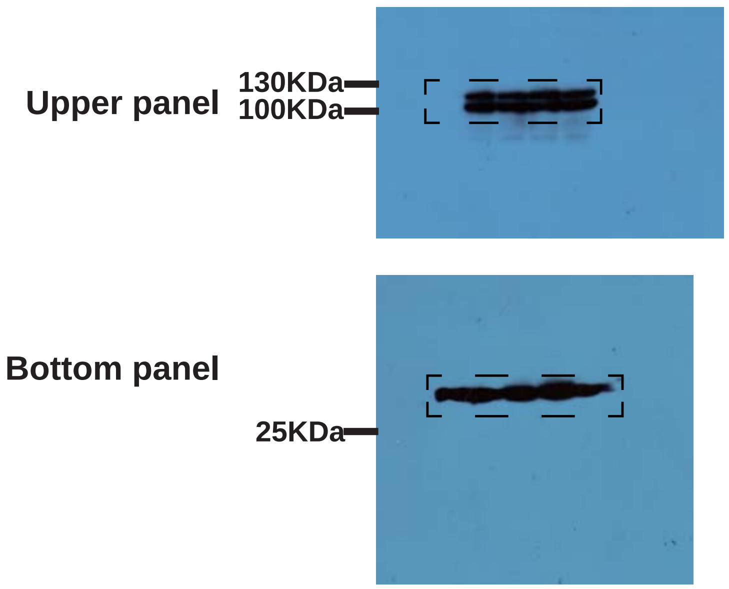

Supplement: Supplementary Information [file srep34174-s1.pdf]
